# Traditional Chinese medicine injection for promoting blood circulation and removing blood stasis in treating angina pectoris of coronary heart disease: A protocol for systematic review and network meta-analysis

**DOI:** 10.1097/MD.0000000000025608

**Published:** 2021-04-23

**Authors:** Langlang Huang, Ri Xu, Xin Huang, Yusa Wang, Jianan Wang, Yanwei Liu, Zhongyong Liu

**Affiliations:** a Jiangxi University of Traditional Chinese Medicine; b The Affiliated Hospital of Jiangxi University of Traditional Chinese Medicine, Nanchang, Jiangxi Province, PR China.

**Keywords:** coronary heart disease, network meta-analysis, protocol, systematic review, traditional Chinese medicine injections

## Abstract

**Background:**

As a common cardiovascular disease, the morbidity and mortality of coronary heart disease (CHD) are increasing year by year. In recent years, many RCTs have proved that compared with conventional therapy, the combination of TCMIs for promoting blood circulation and removing blood stasis can improve clinical efficacy. However, there is still a lack of direct comparative study between different kinds of TCMIs. Therefore, based on the NMA, this study compares the curative effects of various TCMIs for promoting blood circulation and removing blood stasis in treating CHD to provide a reference for clinical medication.

**Methods:**

We will search PubMed, Web of Science, Embase, The Cochrane Library, China National Knowledge Infrastructure, The Chongqing VIP Chinese Science and Technology Periodic Database, Wanfang Database, and China Biomedical Literature Database for the randomized controlled trials of Danhong injection, Xuesaitong injection, Dengzhanxixin injection, and Salvianolate injection in the treatment of CHD, and we will also manually retrieve from the following databases: Chinese Clinical Trial Register, conference papers, and unpublished studies or references. According to the pre-established inclusion and exclusion criteria, 2 researchers independently screened the literature, extracted the data, and evaluated the RCTs’ quality. The primary outcome indicators are the total effective rate of improving angina pectoris symptoms and electrocardiogram improvement. Secondary indicators were angina pectoris attack frequency, angina pectoris attack time, hemorheology, and inflammatory factor level. And use Stata 16.0 software for mesh meta-analysis. Evidence will be checked using the classification of recommendation, evaluation, development, and evaluation.

**Results:**

In this study, from the perspective of different kinds of TCMIs for promoting blood circulation and removing blood stasis, we will compare the curative effects of varying treatment measures and rank the curative effects.

**Conclusion:**

This study will evaluate the efficacy of different kinds of TCMIs for promoting blood circulation and removing blood stasis in the treatment of CHD and help clinicians improve their clinical effectiveness.

**Unique INPLASY number:**

INPLASY202130103.

## 1 Introduction

Coronary heart disease (CHD) refers to heart disease caused by coronary artery atherosclerosis, causing stenosis or occlusion of lumen, leading to myocardial ischemia and hypoxia or necrosis.^[1]^ This disease is the most common type of organ diseases caused by atherosclerosis, which seriously endangers human health. According to the World Health Organization statistics, at present, the death toll of CHD has exceeded the sum of all tumor deaths, becoming the first cause of death.^[2]^ With the rapid development of the social economy and the acceleration of population aging, cardiovascular diseases’ incidence keeps increasing rapidly. Summary of the 2018 report on cardiovascular diseases in China pointed out that there are 290 million people with cardiovascular diseases in China, including 11 million CHDs. The total number of interventional treatment cases of CHD in mainland China is 753,142, with a rapid upward trend in morbidity and mortality, which has brought an enormous economic burden to national medical and health care.^[3]^

Clinically, many patients with CHD often have no apparent symptoms in the early stage. Still, once the condition occurs, severe cases will lead to myocardial infarction and even sudden death. Preventing the occurrence and further development of angina pectoris of CHD has become one of the urgent medical problems. In recent years, due to the rapid growth of medical technology, people turn to revascularization to treat stable angina pectoris of CHD by western medicine. However, while these treatments improve symptoms and prognosis, there are also some problems such as expensive interventional surgery, postoperative restenosis, adverse drug reactions, etc, so preventing thrombosis and restenosis after surgery has become a thorny issue for cardiovascular physicians.^[4,5]^ However, in treating diseases, traditional Chinese medicine (TCM) mainly adopts the principle of syndrome differentiation and treatment, so it is a research topic with practical significance and broad prospects to treat CHD and angina pectoris from the direction of TCM.

At present, traditional Chinese medicine injections (TCMIs) for promoting blood circulation and removing blood stasis have been widely used in the clinical treatment of CHD. According to Chinese medicine, CHD belongs to the category of “Xiongbi” and “Xintong.” Among them, blood stasis syndrome is more common in CHD, which has guiding significance in treating CHD.^[6]^ Moreover, the theory and practice of promoting blood circulation and removing blood stasis in treating CHD have been widely affirmed, and the combination with western medicine can improve the clinical curative effect.^[7]^ From the point of view of western medicine, the fundamental pathological changes of CHD mainly include abnormal blood lipid metabolism, coronary atherosclerotic plaque formation, myocardial ischemia, increased blood viscosity, and hemorheological modifications, which is the embodiment of TCM's understanding of the pathological products of CHD with blood stasis. Studies have shown that the method of promoting blood circulation and removing blood stasis can resist platelet aggregation, remove oxygen free radicals, improve blood circulation, and hemorheology of patients.^[8]^ Promoting blood circulation and removing blood stasis provide a new approach and perspective for CHD treatment and have practical application value.

There are Danhong injection, Xuesaitong injection, Dengzhanxixin injection, and Salvianolate injection for treating CHD. In recent years, several systematic evaluations suggest that combining TCM and western medicine can improve clinical efficacy and effect treating both the symptoms and root causes.^[9–12]^ However, some differences between different studies are different, and there is still no direct comparative study between different kinds of TCMIs. Therefore, in this study, the efficacy of various TCMIs for promoting blood circulation and removing blood stasis on CHD was compared and ranked by network meta-analysis (NMA) to provide a basis for clinical drug selection.

## 2 Methods

### 2.1 Protocol and registration

The protocol has been registered on the INPLASY website (URL https://inplasy.com/inplasy-2021-3-0103/), registration number: INPLASY202130103. This report will be conducted according to the guidance of the Preferred Reporting Items for Systematic Review and Meta-Analysis Protocols (PRISMA-P).^[13]^

### 2.2 Literature search

Two researchers independently searched PubMed, Web of Science, the Cochrane Library, Embase, China National Knowledge Infrastructure, the Chongqing VIP Chinese Science and Technology Periodical Database, Wanfang Database, and China Biomedical Literature Database. The search time is from the establishment of each database to February 2021. We will also manually retrieve from the following databases: Chinese Clinical Trial Register, conference papers, and unpublished studies or references. PubMed's search strategy is shown in Table 1, a similar method will be applied to the Chinese database.

**Table 1:** PubMed search strategy.

Number	Search terms
#1	Coronary Disease[MeSH]
#2	Coronary Diseases[title/abstract]
#3	Disease, Coronary[title/abstract]
#4	Diseases, Coronary[title/abstract]
#5	Coronary Heart Disease[title/abstract]
#6	Coronary Heart Diseases[title/abstract]
#7	Disease, Coronary Heart[title/abstract]
#8	Diseases, Coronary Heart[title/abstract]
#9	Heart Disease, Coronary[title/abstract]
#10	Heart Diseases, Coronary[title/abstract]
#11	Angina Pectoris[MeSH]
#12	Stenocardia[title/abstract]
#13	Stenocardias[title/abstract]
#14	Angor Pectoris[title/abstract]
#15	OR #1–#14
#16	Injections[MeSH]
#17	Injection[title/abstract]
#18	Injectables[title/abstract]
#19	Injectable[title/abstract]
#20	OR #16–#19
#21	danghong[title/abstract]
#22	xuesetong[MeSH]
#23	xuesaitong[title/abstract]
#24	xue sai tong[title/abstract]
#25	OR #22–#24
#26	dengzhanxixin[title/abstract]
#27	Salvianolate[title/abstract]
#28	#21 OR #25 OR #26 OR #27
#29	randomized controlled trial[Publication Type]
#30	randomized[Title/Abstract]
#31	placebo[Title/Abstract]
#32	OR #29–#31
#33	#15 AND #20 AND #28 AND #32

### 2.3 Inclusion criteria

According to the principle of participants, intervention, comparison and outcome (PICO), the following standards are formulated.

(1) *Type of studies*: Randomized controlled trials (RCTs) of treating CHD with traditional Chinese medicine injection (TCMI) for promoting blood circulation and removing blood stasis will be included in this review. The literature language is limited to Chinese and English.
(2) *Type of participants*: Patients diagnosed as CHD according to clinical standards will be included. Gender, age, course of the disease, and other conditions are not limited.
(3) *Types of interventions*: The treatment group included Danhong injection, Xuesaitong injection, Dengzhanxixin injection, and Salvianolate injection combined with routine treatment.
(4) *Type of comparators*: The control group was given routine treatment, which could be combined with placebo or other TCM treatments.
(5) *Types of outcome measures*: This study's primary outcome indicators are the total effective rate of improving angina pectoris symptoms and the improvement of the electrocardiogram. Secondary indicators were angina pectoris attack frequency, angina pectoris attack time, hemorheology (including whole blood viscosity high shear, absolute blood viscosity low shear, red blood cell volume, fibrinogen, blood viscosity), and inflammatory factor level (such as C-reactive protein, IL-6). The safety index is the incidence of adverse reactions.

### 2.4 Exclusion criteria

(1) The control group was treated with TCMI other than intervention measures, and the intervention measure is the study of combining multiple drugs;
(2) Documents with similar original data and repeated publication;
(3) Research on incomplete data reporting, duplicate data, or inability to extract data;
(4) Animal experimental literature;
(5) Case report, conference papers, and summaries;
(6) CHD with other complications;
(7) No randomized controlled trial (RCT).

### 2.5 Studies selection

**Figure 1:**
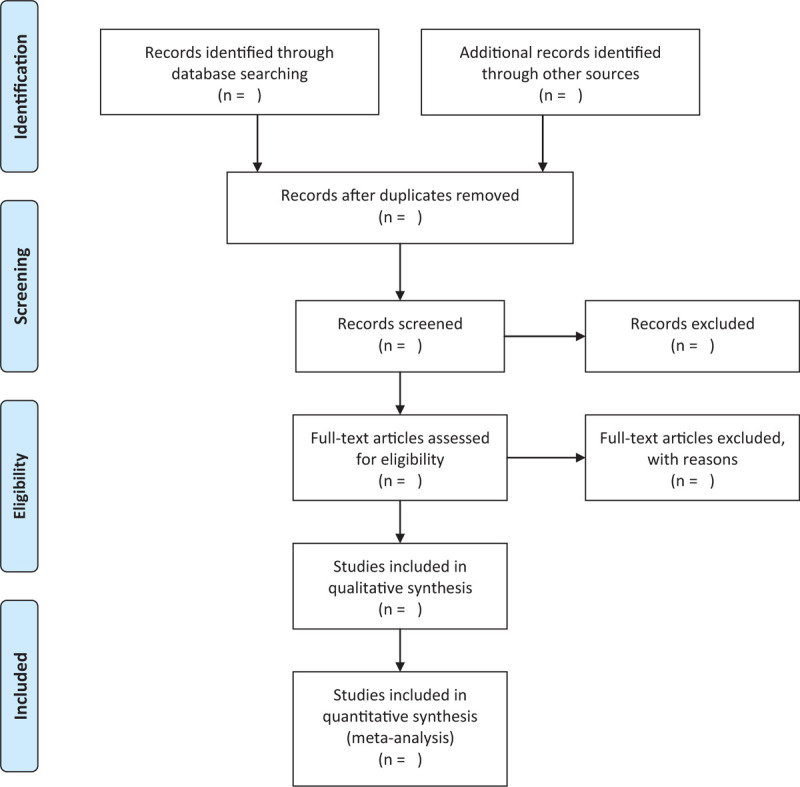
Flow diagram of literature retrieval.

Two researchers independently screened the literature according to the established screening criteria. Find duplicate documents through Endnote X9 document management software, and check them manually; read the title and abstract of the literature, and after screening according to the inclusion criteria, download the full text, and read it to exclude further the literature that does not meet the inclusion criteria. If there is disagreement, the third author discussed and decided together. The selection process will be shown according to the PRISMA flow chart in Figure 1.

### 2.6 Data extraction and management

After the literature was included, 2 researchers used the pre-established data extraction table to extract the data of the included studies, respectively, including the author's name, year of publication, title, country, average age, gender, study design, the total number of cases, participants, intervention measures, comparison, outcome, and any other relevant information.

### 2.7 Quality assessment of the studies

According to the bias risk evaluation standard of randomized controlled trials provided by Cochrane Handbook,^[14]^ the literature quality evaluation is carried out, which includes the following 7 aspects: random method, assignment concealment, blind method of subjects, blind method of result evaluation, data integrity, selective report, and other bias. According to the specific scoring rules, the 2 researchers evaluated 3 types: “low risk,” “high risk,” and “uncertain risk.” Researchers independently complete and crosscheck, and if there are differences, they will be resolved through discussion or assistance by a third researcher.

### 2.8 Measures of treatment effect

In this study, the total effective rate is a dichotomous variable, and the odds ratio (OR) is used as the effect quantity, and the corresponding 95% confidence interval (CI) is calculated. For other outcome indicators, such as measurement data, the mean difference (MD) is selected as the effect analysis statistic, and its effective value and 95% CI are provided.

### 2.9 Dealing with missing data

If the data is insufficient or lost, we will first contact the original author by email or telephone. If the lost data cannot be obtained, we will discard the unusable data and only analyze the available information.

### 2.10 Assessment of heterogeneity

If there is no heterogeneity among the research results (*P* > .10, *I*^2^ < 50%), the fixed effect model is used for meta-analysis. If heterogeneity (*P* ≤ .10, *I*^2^ ≥ 50%), the random effect model will be used for meta-analysis after the apparent clinical heterogeneity is excluded.

### 2.11 Statistical analysis

The software Revman 5.3 and Stata 16.0 were used for data analysis. RevMan 5.3 was used to evaluate the quality of the included literature. Stata 16.0 software is used for direct meta-analysis and network meta-analysis based on frequency framework. In the NMA, the network group command is used to preprocess the data, the network evidence diagram and the “comparison-correction” funnel diagram are drawn, the different interventions are compared in pairs, and the optimal probability ranking curve surface under the cumulative ranking curve (SUCRA) is calculated to rank the efficacy.^[15]^ The higher the SUCRA value, the better the intervention level in the network. A SUCRA value of 100% indicates that the treatment is the most effective in the network. An inconsistency check is performed when there is a closed loop. If there is evidence of direct comparison and indirect comparison at the same time, that is, there are closed loops, the consistency of each closed loop is evaluated by using the inconsistency factor (IF) and its 95% CI, and 95% CI contains 0 as good consistency; otherwise, it is considered that the closed loop has obvious inconsistency.

### 2.12 Summary of evidence

The evidence quality of each result is evaluated by the method of Recommendation, Assessment, Development, and Evaluation (GRADE).^[16]^ The evaluation will be divided into 4 qualities: “very low,” “low,” “medium,” or “high.”

### 2.13 Ethics and dissemination

Ethical approval is not required for this study because there is no individual data will be used. The research results will be published in a peer-reviewed journal and conference presentations.

## 3 Discussion

TCM has a long history of thoracic obstruction and heartache, which has accumulated rich clinical experience and formed an independent theoretical system. Blood stasis blocking heart pulse is the main pathological link of CHD, so promoting blood circulation and removing blood stasis can improve patients’ clinical symptoms and prognosis. Danhong injection, Xuesaitong injection, Dengzhanxixin injection, and Salvianolate injection are TCMs for promoting blood circulation and removing blood stasis, widely used in the clinical treatment of CHD.^[17–20]^ Danhong injection is made from Salvia miltiorrhiza and Carthamus tinctorius L, which has anti-inflammatory, anti-oxidation, anti-coagulation, improving blood rheology, reducing blood lipid, resisting atherosclerosis, promoting angiogenesis, protecting vascular endothelium, resisting apoptosis, resisting platelet aggregation, and improving microcirculation.^[21,22]^ Xuesaitong injection is made from total saponins of panax notoginseng (PNS), which is the main active component of PNS. Basic research shows that Xuesaitong injection can significantly reduce the peripheral resistance of blood vessels, dilate coronary arteries, relax vascular smooth muscle, increase myocardial oxygen supply, and improve myocardial ischemia.^[23]^ Breviscapine is an effective active component of flavonoids extracted from Dengzhanxixin, which can scavenge free radicals, inhibit inflammatory factors, resist platelet aggregation, and relax blood vessels.^[24,25]^ Salvianolic acid is the main component of Salvia miltiorrhiza, TCM for promoting blood circulation and removing blood stasis. Studies have shown that it can stabilize atherosclerotic plaque, inhibit platelet aggregation, improve vascular endothelial function, and resist lipid peroxidation.^[26]^

The application of NMA to correlate the meta-research is beneficial to the clinical evaluation of many similar TCMIs and provides evidence support for clinical rational drug use. Therefore, we hope that this study can provide the latest evidence for the effectiveness and safety of TCMI in treating CHD and the difference of curative effect, and can be used to guide clinical practice.

## Author contributions

**Conceptualization:** Langlang Huang, Yanwei Liu, and Zhongyong Liu.

**Data curation:** Langlang Huang, Jianan Wang, and Ri Xu.

**Formal analysis:** Yusa Wang, Xin Huang, and Yanwei Liu.

**Investigation:** Langlang Huang, Yanwei Liu, and Zhongyong Liu.

**Methodology:** Yusa Wang, Jianan Wang, Ri Xu, and Xin Huang.

**Software:** Yusa Wang, Xin Huang, and Jianan Wang.

**Supervision:** Zhongyong Liu and Yanwei Liu.

**Writing – original draft:** Langlang Huang, Xin Huang, Jianan Wang, and Zhongyong Liu.

**Writing – review & editing:** Langlang Huang, Ri Xu, Yusa Wang, and Yanwei Liu.
